# Low-Dose Intratympanic Gentamicin Injections for Intractable Meniere’s Disease: How Many Are Optimal?

**DOI:** 10.3390/jcm14124342

**Published:** 2025-06-18

**Authors:** Joon-Pyo Hong, Hayoung Byun, Min-Beom Kim

**Affiliations:** 1 Department of Otorhinolaryngology-Head and Neck Surgery, Kangbuk Samsung Hospital, Sungkyunkwan University School of Medicine, Seoul 03181, Republic of Korea; enthjp@gmail.com; 2 Air Defense Control & Command, 15th Fighter Wing, Gyeonggi-do 13102, Republic of Korea; 3 The Best Care ENT Clinic, Seoul 02559, Republic of Korea

**Keywords:** Meniere’s disease, gentamicin, dizziness

## Abstract

**Background/Objectives:** To compare the outcomes of low-dose intratympanic gentamicin injection (ITGM) in managing intractable Meniere’s disease (MD) between patients receiving a single injection versus multiple injections, and to explore the optimal number of ITGM repetitions. **Methods:** This retrospective study was conducted at a single tertiary medical center. Clinical charts of patients diagnosed with definite MD between 2015 and 2020 and given low-dose ITGM for intractable vertigo attacks were reviewed. A total of 33 patients were divided into two groups based on the number of ITGM procedures: the single injection group (SG, n = 14) and the multiple injection group (MG, n = 19). In the MG, additional ITGM was performed up to four times. Audiograms, caloric responses, and video head impulse tests (vHIT) were reviewed at each repetition of ITGM. **Results:** After the first ITGM, both the SG and MG showed significant decreases in caloric responses and vHIT gains, without deterioration in hearing. In the MG group, a second ITGM was needed on average 8.1 ± 6.4 months after the initial ITGM due to persistent vertigo attacks. After the second ITGM, 8 out of 19 MG patients showed additional benefits in terms of reduced vertigo and further decreases in caloric responses. However, after the third and fourth ITGM, no further significant decline in vestibular function was observed, and there was no improvement in subjective dizziness. In the MG, gradual deterioration of hearing was observed. **Conclusions:** This finding suggests that performing additional low-dose ITGM in poorly responding or recurrent cases appears reasonable up to the second injection. For those who continue to experience vertigo episodes after two ITGM procedures, alternative therapeutic approaches should be considered to preserve hearing.

## 1. Introduction

Ménière’s disease (MD) is a clinical syndrome characterized by recurrent vertigo and fluctuating aural symptoms (hearing loss, tinnitus, or ear fullness) [1]. A recent revision in the diagnostic criteria for MD by the Classification Committee of the Bárány Society has simplified MD into two categories: probable MD and definite MD [2]. This change has reduced the complexity of clinical diagnosis but we still do not fully understand the exact etiology of MD, and its treatment remains challenging [3]. Although endolymphatic hydrops is one of the most widely recognized pathophysiologies, there are several other potential mechanisms of MD, including manifestations of inner ear migraine [4,5,6].

Intratympanic gentamicin injection (ITGM) is a recommended treatment option for intractable MD that does not respond to noninvasive treatments [1]. Because gentamicin is more toxic to vestibular hair cells than to cochlear hair cells, it is widely used in intractable MD to chemically ablate peripheral vestibular sensors from the endolymphatic hydrops attack in the inner ear [7,8]. Moreover, the gentamicin also primarily targets type I vestibular hair cells, which are more susceptible than type II cells due to their greater absorption and retention of the drug [7]. This selective toxicity underlies its use in treating intractable vertigo, as recurrent episodes in Meniere’s disease are thought to be more closely associated with dysfunction of type I cells [7]. However, consensus is lacking regarding the appropriate ITGM protocol and a reliable vestibular test battery [1,9]. In addition to its well-established impact on quality of life [10], intractable MD also demonstrates significant correlations with various clinical parameters, such as vestibular function test results, hearing thresholds, and the frequency of vertigo attacks [1]. These associations underscore the importance of comprehensive assessment in determining the optimal treatment strategy of ITGM intervention [1].

Herein, we reviewed patients diagnosed with definite MD who underwent low-dose ITGM due to intractable vertigo attacks after conservative treatment. We assessed the clinical data of audiometric evaluation and vestibular tests of these patients. A comparison between the single injection group (SG) and multiple injection group (MG) was performed to explore the rationale for determining the optimal number of ITGM repetitions.

## 2. Materials & Methods

### 2.1. Subjects

**Figure 1:**
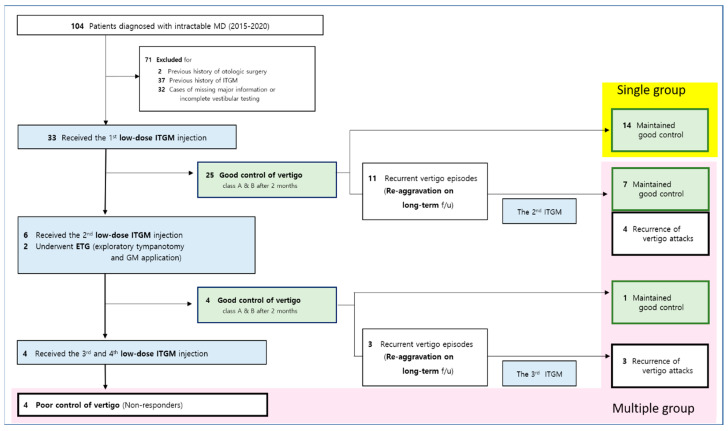
Flowchart of the study. MD (Ménière’s disease); ITGM (intratympanic gentamicin injection).

We conducted a retrospective medical chart review of patients diagnosed with definite MD at our tertiary medical center between 2015 and 2020 (Figure 1). Definite Ménière’s disease was diagnosed based on the following established criteria: 1. two or more spontaneous episodes of vertigo, each lasting between 20 min and 12 h. 2. audiometrically confirmed fluctuating low- to mid-frequency sensorineural hearing loss in the affected ear on at least one occasion before, during, or after a vertigo episode. 3. fluctuating aural symptoms, such as hearing loss, tinnitus, or aural fullness, in the affected ear. 4. exclusion of other potential causes through appropriate diagnostic testing [1]. Of them, those who underwent low-dose ITGM (n = 33) due to intractable vertigo were included in this study (Figure 1). Intractability was defined by vertigo attacks that were unresponsive to lifestyle modifications and oral medications (betahistine and/or diuretics) for at least 6 months, based on the previous studies of intractable MD [8,11,12,13,14]. None of the included patients had previously received surgical intervention or ITGM. A history of treatment with medications other than gentamicin, including steroids, for Ménière’s disease was not considered an exclusion criterion. Every included patient underwent audiometric evaluation and vestibular tests before the initial ITGM to check a baseline function and two months after each ITGM trials. Good response was defined as better than Class B vertigo control; that is, greater than a 60% reduction or less than 40% of the baseline vertigo episode severity, (1) for a minimum of six months following the ITGM trials, without any further request for interventions by the patients [11,14,15]. If vertigo spells recurred within or after six months of each ITGM session, additional sessions were administered, up to a maximum of four trials. Patients who experienced acute spontaneous nystagmus with prominent catch-up saccades immediately after ITGM were considered to have undergone a successful procedure and those who later reported persistent tipsy disequilibrium were not considered for additional ITGM, in order to distinguish successful ablation from intractable vertigo spells [15].

### 2.2. Audiometric Tests

The hearing thresholds of four frequencies (500, 1000, 2000, and 3000 Hz) in the pure tone audiometry (PTA) were evaluated. The average threshold was determined by taking the mean of the hearing levels across the four frequencies, with each given equal weight [13,14,16,17]. Pure-tone audiometry was conducted using an audiometer (Interacoustics, Denmark) in a dedicated soundproof booth specifically designed for audiological testing. To accurately evaluate the effect of ITGM on hearing, all patients underwent baseline audiometric evaluation prior to ITGM administration. This pre-treatment assessment allowed for a reliable comparison of hearing thresholds before and after the intervention. The word recognition score (WRS) was assessed using standard Korean monosyllabic word lists [18]. All audiometric evaluations were performed one month after each ITGM trial.

### 2.3. Vestibular Tests

Video head impulse test (vHIT) and caloric response test were conducted as vestibular tests. For the vHIT, a high-frame video-oculography device (ICS Impulse, Otometrics, Denmark) was utilized to record gains across all three semicircular canals (SCCs). Gains were calculated using the ratio of the amplitude of head impulse velocity to slow phase velocity of the eyes. During the recordings, the peak head velocity of the impulses was carefully controlled, maintained between 150 degrees/second and 250 degrees/second, using visual feedback of the head movement [19]. At least 20 valid head impulse responses were obtained for each SCC. A normal gain was defined as >0.8 for the horizontal canals and >0.7 for the vertical canals [19]. All vHIT examinations were performed by a single designated examiner to minimize inter-operator variability and reduce potential measurement errors. The caloric test was performed to assess the function of the horizontal semicircular canal at low frequencies. Canal paresis (CP) was defined as a unilateral weakness of ≥30%, based on criteria used in previous studies conducted at our institution [19,20]. For the caloric response test, bithermal water irrigation stimulation with temperatures of 44 °C and 30 °C (Micromedical Technologies Inc., Chatham, IL, USA) was performed. A CP was quantified according to the Jongkee formula [20]. All vestibular tests were performed one month after each ITGM trial.

### 2.4. Low-Dose Intratympanic Gentamicin Injection (ITGM) Administration

The tympanic membrane was anesthetized using a 1 × 1 cm gauze soaked in lidocaine for 10 to 20 min. A low-dose (20 mg/mL) gentamicin solution was prepared by mixing 0.5 mL of gentamicin sulfate (40 mg/mL) with 0.5 mL of saline. This solution was then injected into the mid-posterior aspect of the anesthetized tympanic membrane. Patients were instructed not to swallow or speak, and they were required to remain in a supine position. The head was angled slightly downward and turned to the contralateral side for 30 min [21]. The same dosage and method of ITGM were applied to all patients. Injections were performed under direct visualization using an ear endoscope for accurate delivery.

### 2.5. Exploratory Tympanotomy

A tympanomeatal flap was elevated and flipped superiorly to allow access to the middle ear cavity. Under microscopic visualization, the accessible middle ear space was meticulously examined for any anatomical obstructions, such as fibrotic webs or scar tissue, that might impede access to the round window niche. Subsequently, a 30-degree endoscope was employed to provide enhanced visualization of the round window region. Gelfoams soaked in a low-dose gentamicin solution (20 mg/mL) were carefully placed in proximity to the round window. After completion of the procedure, the tympanomeatal flap was repositioned, and the operation was ended.

### 2.6. Statistical Analysis

The Student’s *t*-test was used to compare the mean age and the period from diagnosis to the first ITGM between the SG and MG. The Chi-square test and Fisher’s exact test were used to examine the differences in distribution of gender and lesion site between the SG and MG. A paired *t*-test was utilized to compare the audiometric and vestibular test results between each pre- and post-ITGM trial in the SG and MG. The normality of the data distribution was assessed using the Shapiro–Wilk test. Continuous variables are presented as means and standard deviations. All statistical analyses were conducted using SPSS version 24.0 for Windows (SPSS Inc., Chicago, IL, USA) and a 5% significance level was considered.

## 3. Results

### 3.1. Patient Characteristics

Among the total of 33 patients, 14 and 19 patients were categorized into SG and MG, respectively (Figure 1). The mean age of patients in the SG was 56.7 ± 14.6 years, while in the MG it was 56.8 ± 11.6 years. The mean period from diagnosis to the first ITGM was 3.4 ± 2.8 years for the SG and 2.5 ± 1.4 years for the MG. For the MG, the average durations between the first and second ITGM, second and third ITGM, and third and fourth ITGM were 8.1 ± 6.4 months, 20.4 ± 11.5 months, and 3.4 ± 2.5 months, respectively. Of the 19 patients in MG, 11 underwent a third ITGM, and 3 of these 11 underwent a fourth ITGM. There were no significant differences in mean age, the mean period from diagnosis to the first ITGM, distribution of gender and lesion site between the SG and MG, supporting the statistical comparability of the two groups at baseline (Table 1). Every patient had an intact vestibular function in the contralesional ear.

### 3.2. Audiometric Evaluation in Single Injection Group (SG) and Multiple Injection Group (MG)

**Figure 2:**
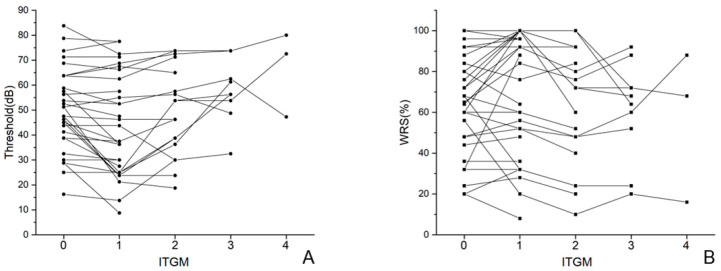
Changes in average hearing threshold (**A**) and WRS (**B**) for each patient. Each patient is represented by a black dot in the graphs. WRS (word recognition score; ITGM (intratympanic gentamicin injection).

In the SG, average hearing thresholds after the single ITGM significantly improved from 51.3 ± 16.7 dB to 46.6 ± 20.7 dB (*p* = 0.040), while the WRS showed no significant difference (from 66.3 ± 29.2% to 65.4 ± 30.7%, *p* = 0.872). In the MG, average hearing thresholds after the first ITGM improved from 50.3 ± 15.9 dB to 42.5 ± 19.4 dB (*p* = 0.005) while the WRS showed no significant difference (from 66.8 ± 22.2% to 75.6 ± 27.9%, *p* = 0.052). Comparing results between the first and second ITGM, average hearing thresholds significantly worsened from 42.5 ± 19.4 dB to 49.7 ± 17.4 dB (*p* = 0.008) and the WRS also significantly worsened from 75.6 ± 27.9% to 66.4 ± 28.6% (*p* = 0.003). Comparing the second and third ITGM for those who proceeded to third trials (n = 11), the average hearing threshold worsened from 51.7 ± 14.4 dB to 58.0 ± 11.4 dB (*p* = 0.047) and the WRS showed no significant difference (from 66.4 ± 30.6% to 62.2 ± 22.9%, *p* = 0.459) (Figure 2).

### 3.3. Vestibular Test Results in Single Injection Group (SG) and Multiple Injection Group (MG)

**Figure 3:**
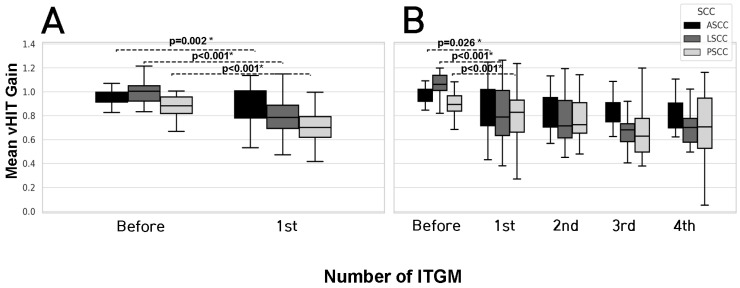
Gain changes of SG and MG. Significant decreases were observed only after the first injection in both SG (**A**) and MG (**B**). Only statistically significant *p*-values are shown. (* *p* < 0.05). The absence of a comparison line indicates no statistical significance; *p* > 0.05. vHIT (video head impulse test); SG (single injection group); MG (multiple injection group); SCC (semicircular canals); ASCC (anterior semicircular canal); LSCC (lateral semicircular canal); PSCC (posterior semicircular canal); ITGM (intratympanic gentamicin injection).

In the SG, the initial gain of the anterior, lateral, and posterior SCCs was 0.95 ± 0.13, 1.01 ± 0.12, and 0.91 ± 0.17, respectively. After the single injection, the gain of the anterior, lateral, and posterior SCCs was significantly decreased to 0.72 ± 0.22 (*p* = 0.002), 0.63 ± 0.21 (*p* < 0.001), and 0.59 ± 0.18 (*p* < 0.001), respectively. In the MG, the initial gain of the anterior, lateral, and posterior SCCs was 0.96 ± 0.07, 1.03 ± 0.10, and 0.88 ± 0.12, respectively. After the first injection, the gain of anterior, lateral, and posterior SCCs was also significantly decreased to 0.87 ± 0.17 (*p* = 0.026), 0.81 ± 0.23 (*p* < 0.001), and 0.71 ± 0.25 (*p* < 0.001), respectively, while remaining mostly within normal limits despite the significant decrease. After the first injection, no further significant decreases in all three SCCs were identified. There were no significant differences in all SCC gain values between the SG and MG groups, either before or after the initial ITGM (Figure 3).

**Figure 4:**
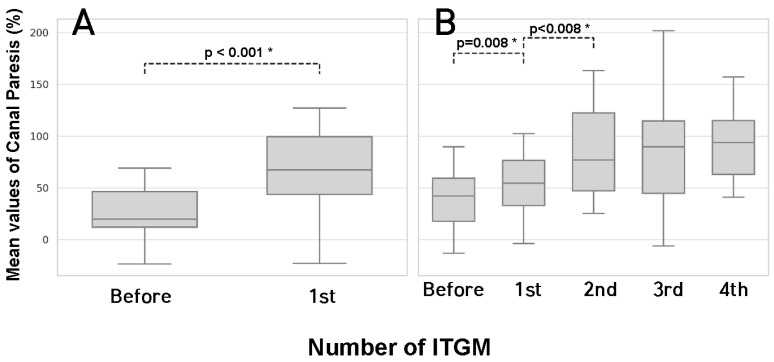
Caloric response test results of SG and MG. Significant increases in canal paresis were observed after the first ITGM in both SG (**A**) and MG (**B**). An additional significant increase in canal paresis was observed after the second ITGM in MG (**B**). Only statistically significant *p*-values are shown. (* *p* < 0.05). The absence of a comparison line indicates no statistical significance; *p* > 0.05. SG (single injection group); MG (multiple injection group); ITGM (intratympanic gentamicin injection).

In the SG, the initial CP from the caloric response test was 25.14 ± 22.68%. After the single injection, the CP was significantly increased to 78.07 ± 24.06% (*p* < 0.001). In the MG, the initial CP from the caloric response test was 29.84 ± 16.11%. After the first injection, the CP was significantly increased to 52.00 ± 31.55% (*p* = 0.008) and after the second injection, the CP was further significantly increased to 80.68 ± 26.20% (*p* < 0.001). However, no additional significant increases in the CP were observed after the second injection. As with the vHIT findings, there were no significant differences in CP values between the SG and MG groups, either before or after the initial ITGM (Figure 4).

## 4. Discussion

In this study, we compared the SG and MG in terms of hearing thresholds, vestibular test results, and vertigo recurrence. In the SG, patients experienced relief from vertigo after a single ITGM attempt, accompanied by significant decreases in both the vHIT and caloric response tests. In contrast, although patients in the MG group also exhibited significant vestibular loss after the initial ITGM, they continued to suffer from recurrent vertigo episodes and required additional ITGM treatments. After the second ITGM, a further decrease in caloric responses was observed and 8 out of 19 MG patients achieved symptomatic relief. However, in subsequent ITGM trials more than two the number of ITGM injections did not yield additional evidence of vestibular ablation and were associated only with progressive hearing deterioration.

The role of ITGM in MD has consistently received attention. It has been considered as a useful treatment for intractable vertigo spells in MD by reducing the sensitivity of the peripheral vestibular organs [1,22,23]. Since ITGM is an invasive procedure involving chemical vestibular ablation, yet plays a crucial role in the management of intractable Ménière’s disease at the same time, a unified consensus on its use in treating intractable MD is needed.

Ototoxicity from aminoglycoside is one of the main concerns in ITGM [24,25]. However, the safety of low-dose gentamicin has been proposed, with potential benefits in hearing preservation and vertigo control [26,27,28]. In this study, within the SG, there was an observed advantage of low-dose ITGM in improving hearing thresholds. The SG consisted of patients who experienced significant resolution in vertigo attacks after a single ITGM treatment. Thus, we can hypothesize that patients in the SG may have individual advantages regarding drug delivery to the inner ear, possibly due to a low anatomical barrier around the round window or enhanced permeability of the round window membrane [29,30]. Considering the balance between the controllability of the disease through ITGM and the progression of intractable MD, the improvement in the SG’s hearing could be explained by the disease control efficacy outweighing the disease progression. A prominent impairment in vestibular tests within the SG support this hypothesis, as previous studies have shown that distinct impairments reflect the degree of chemical ablation in vestibular organs [31,32]. However, while this hypothesis provides a plausible explanation, we did not directly measure drug delivery rates or anatomical differences in the round window membrane. Therefore, the more favorable response observed in the SG should be interpreted with caution. It remains equally, if not more, likely that the observed differences may reflect heterogeneity in disease phenotype or individual susceptibility to gentamicin, rather than differences in drug delivery efficiency alone.

Interestingly, in the initial phase, the MG displayed vestibular test results similar to those observed in the SG. Like the SG, the MG also demonstrated improvements in the hearing thresholds and significant impairments in vestibular tests following the first ITGM. Therefore, as mentioned above, we cannot discount the possibility that those requiring multiple injections of ITGM also experienced sufficient delivery of gentamicin into the inner ear following the initial injection. The only discernible difference between the SG and MG after the initial ITGM was the continuation of subjective vertigo attacks, with an average gap of 8.1 ± 6.4 months between the initial and second ITGM. While a previous report suggested that a second ITGM could be avoided if there was more than a 17.8% gain reduction in the lateral SCC as evaluated by the vestibulo-ocular reflex [33], both the SG and MG in the current study exhibited even greater gain reductions (37.6% for the SG and 21.4% for the MG, respectively). Since a major reduction in gain typically occurs around 3 to 4 weeks after the initial ITGM [34], the two-month period between the ITGM and the vestibular test follow-up in this study seems sufficient. In addition, there were no significant differences in SCC gain or caloric response test results between the SG and MG groups, either before or after the initial ITGM. This suggests that patients in the MG may also have received sufficient drug delivery during the initial ITGM trial. Therefore, this result makes us unable to identify any objective factors that could predict recurrence during follow-up among successful ITGM cases who showed good responses after the first ITGM. If patients continue to experience recurrent vertigo attacks despite evidence of prominent vestibular loss after ITGM, an additional injection trial may be considered carefully.

After the second ITGM, hearing thresholds in the MG appeared to progressively worsen. Moreover, no additional vestibular impairments were evident in the vestibular test results after the second ITGM, with the sole exception being the CP observed in the caloric response test after the second ITGM. Although CP is considered less reliable than gain in predicting the effects of gentamicin [35], caloric test results should not be complacently overlooked. They may still indicate the potential for additional vestibular ablation, especially given the absence of quantitative differences between the SG and MG groups after the initial ITGM attempt. Additionally, considering that 8 out of 19 patients in the MG achieved relief from intractable MD after the second ITGM, we suggest that a second ITGM attempt may be reasonable in cases where patients continue to experience recurrent vertigo spells after the initial treatment, even in the presence of prominent vestibular function deterioration. However, continuing ITGM beyond two attempts appears to provide no additional benefit and is associated only with progressive hearing loss, especially declines in WRS.

Therefore, we interpret these results to suggest that the need for more than two ITGM treatments is not attributable to insufficient chemical ablation of the peripheral vestibular organs. Additionally, the deterioration of hearing in the MG might be attributed to the progression of the disease independent of the sufficient delivery of gentamicin to the inner ear after ITGM. While cumulative inner ear toxicity from repetitive ITGM is a potential concern, the safety of low-dose ITGM with respect to hearing has been consistently reported. Therefore, caution is warranted in interpreting these findings [9,24,36].

It is noteworthy that two patients from the MG underwent exploratory tympanotomy to identify and remove anatomical barriers in the middle ear, and to place gentamicin-soaked gelfoam near the round window [29,37]. Nevertheless, neither of these two patients demonstrated any evidence of adhesive blockage near the round window or a thickened round window membrane intraoperatively, with failure of vertigo control even after the gentamicin-soaked gelfoam deposit. Given these observations—the satisfactory vestibular deterioration after the first ITGM in the MG and the two unsuccessful exploratory tympanotomy cases—also suggest that drug delivery alone might not fully account for the limitations of ITGM.

There are several possible explanations. First, personal factors such as an individual’s susceptibility to gentamicin might influence ITGM outcomes [38,39]. In other words, the key to successful treatment in cases of intractable MD that do not respond to more than two ITGM attempts may lie in an individual’s capacity to recover from, or resist, repeated gentamicin exposure. The second possible explanation involves the distinct pathophysiology of intractable MD. Instead of endolymphatic hydrops attacks, mechanisms independent of endolymphatic flow, such as inner ear migraines, might be more applicable [4,5].

This study has several limitations. First, as we reviewed patients over 5 years (from 2015 to 2020), there exists a potential for further progression of MD in the SG. Moreover, the presence of long inter-critical periods in MD may confuse the interpretation of symptom control, as temporary remission could be mistaken for treatment success. Second, given the small sample size in this study (a total of 33 patients), there is a need for future studies with larger patient populations. Third, this study did not include detailed documentation of the number, intensity, or characteristics of vertigo episodes—such as Tumarkin’s otolithic crises—which limits our ability to fully characterize treatment outcomes and patient-reported symptom profiles. Fourth, we could not classify patients based on vestibular- or cochlear-onset types, bilaterality, or potential genetic predispositions, which may influence treatment response and disease progression in meticulous ways.

## 5. Conclusions

By exploring symptom outcomes and vestibular test results in patients with intractable MD, our findings suggest that administering additional low-dose ITGM is reasonable up to the second injection for cases with persistent vertigo attacks. For those who continue to experience vertigo episodes thereafter, alternative therapeutic approaches should be considered due to the limited benefit and the risk of ototoxic hearing loss.

## Figures and Tables

**Table 1:** Demographics of the patients in the single injection group and the multiple injection group.

	Single Injection Group (N = 14)	Multiple Injection Group (N = 19)	*p* Value
Age (years)	56.7 ± 14.6	56.8 ± 11.6	0.489
Gender			
Male/Female	6 (42.9%)/8 (57.1%)	6 (31.6%)/13 (68.4%)	0.716
Site			
Right/Left	11 (78.6%)/3 (21.4%)	10 (52.6%)/9 (47.4%)	0.160
Initial average hearing threshold (dB)	51.3 ± 16.7	50.3 ± 15.9	0.873
Initial WRS (%)	66.3 ± 29.2	66.8 ± 22.2	0.955
Initial vHIT gains			
ASCC	0.95 ± 0.13	0.96 ± 0.07	0.763
LSCC	1.01 ± 0.12	1.02 ± 0.1	0.576
PSCC	0.91 ± 0.17	0.88 ± 0.12	0.591
Initial canal paresis (%)	25.1 ± 12.7	27.8 ± 16.1	0.734
Period from diagnosis to first ITGM	3.4 ± 2.8 years	2.5 ± 1.4 years	0.541
Period from first to second ITGM		8.1 ± 6.4 months	
Period from second to third ITGM		20.4 ± 11.5 months	
Period from third to fourth ITGM		3.4 ± 2.5 months	

Student’s *t*-test was used for age, initial average hearing threshold, initial WRS, initial vHIT gains, and period from diagnosis to first ITGM; Chi-square test or Fisher’s exact test was applied for gender and lesion site distribution. Statistical significance was set at *p* < 0.05. Abbreviations: WRS (word recognition score); vHIT (video head impulse test); ASCC (anterior semicircular canal); LSCC (lateral semicircular canal); PSCC (posterior semicircular canal); ITGM (intratympanic gentamicin injection). Values are shown as mean ± standard deviation.

## Data Availability

The datasets used and/or analyzed during the current study are available from the corresponding author on reasonable request.

## Abbreviations

MD	Meniere’s disease
ITGM	Intratympanic gentamicin injection
SG	Single injection group
MG	Multiple injection group
PTA	Pure tone audiometry
WRS	Word recognition score
vHIT	Video head impulse test
SCCs	Semicircular canals
CP	Canal paresis
